# Effectiveness of adjuvant systemic therapy following complete cytoreductive surgery in patients with recurrent granulosa cell tumours of the ovary

**DOI:** 10.1038/s41598-024-51752-x

**Published:** 2024-01-10

**Authors:** Harika Yumru Celiksoy, Catriona Dickie, Michael J. Seckl, Esra Aydın, Hamdullah Sozen, Samet Topuz, Christina Fotopoulou

**Affiliations:** 1 Department of Gynaecologic Oncology, Istanbul University Faculty of Medicine, Istanbul, Turkey; 2 Department of Gynaecologic Oncology, Imperial College London Faculty of Medicine, London, UK; 3 Department of Medical Oncology, Imperial College London Faculty of Medicine, London, UK; 4 Department of Medical Oncology, Istanbul University Faculty of Medicine, Istanbul, Turkey

**Keywords:** Chemotherapy, Surgical oncology

## Abstract

Aim of the present analysis is to compare the impact of antihormonal therapy versus cytotoxic chemotherapy versus a watch a wait approach on disease-free survival (DFS) in the adjuvant setting of patients who underwent complete cytoreductive surgery(CRS) for recurrent adult type granulosa cell tumours of the ovary (GCT). Moreover, we wished to identify prognostic risk factors for recurrence. We included recurrent GCT-patients who underwent CRS resulting in total macroscopic tumour clearance, treated in two gynaecological cancer centres over a 20-year period (2000–2020). CRS was performed for 51 recurrences in 26 GCT-patients. Adjuvant systemic treatments were as follows: chemotherapy in 21 cases, hormonotherapy in 10 cases, no systemic treatment in 20 cases. There were no statistically significant differences in DFS between chemotherapy, hormonotherapy and no systemic treatment: median DFS was 57, 36 and 57 months, respectively (*p* = 0.616). Extra-pelvic and/or multifocal tumour dissemination were found to be independent predictive factors for subsequent recurrences. In the cases with both lower and upper abdominal involvement (n = 18), patients who received chemotherapy (n = 9) had longer DFS than those who had hormonotherapy (n = 2) or no adjuvant therapy (n = 7) at all: median DFS was 36, 13 and 15 months, respectively (*p* = 0.9). Our findings do not encourage the administration of adjuvant therapy following complete CRS for GCT-relapse. Selected high-risk patients with disseminated disease may derive clinical benefit from additional chemotherapy, larger-scale multicentre studies are warranted to define treatment algorithms for this rare disease.

## Introduction

Adult granulosa cell tumours of the ovary (GCT) are the most frequent malignant sex cord-stromal tumours and yet comprise less than 5% of cases of ovarian cancer^1^. GCTs are typically associated with a favorable prognosis, but they can recur years later with a multi-visceral dissemination pattern representing a therapeutic dilemma. When feasible, cytoreductive surgery, equivalent to their epithelial counterparts, is considered as the cornerstone of treatment in both the primary and the recurrent setting. Numerous authors have described the feasibility and success of multiple cytoreductive attempts in chronic disease^2^. With a response rate of 63% to 80%, platinum-based chemotherapy is presently used for patients with advanced or recurrent disease and is also advised for adjuvant postoperative chemotherapy. There is substantial reason for a hormone-based strategy given the functional hormonal character of granulosa cell tumors that express steroid hormone receptors. Several case series have shown that responses to gonadotropin-releasing hormone agonists (GnRH) , tamoxifen, progestins, and aromatase inhibitors have resulted in limited effects, partial responses, disease stability, or no benefit from therapy. This treatment has an acceptable toxicity profile and is simple to administer (oral)^3,4^. Nevertheless, treatment options following maximal cytoreduction are not well defined with strong variation in practice internationally between cancer centres and healthcare systems. Management options range from only watch and wait to antihormonal treatment or even cytotoxic chemotherapy, depending on local practice, experience and clinical governance^5,6^.

Limited information exists on the comparative value of the various adjuvant treatment options after complete cytoreductive surgery. We wished to explore exactly that and assess the impact of the various adjuvant systemic treatment alternatives in GCT-patients following complete cytoreduction for recurrence as well as to define risk factors for further recurrence.

## Methods

After obtaining local ethical approvals (institution review board number E-29624016-050.99-518857) and confirming that all methods were performed in accordance with the relevant guidelines and regulations, we conducted a retrospective cohort analysis of all consecutive patients who underwent complete surgical cytoreduction for GCT-relapse within a 20 years period (2000–2020 in two large tertiary referral centres for gynaecological malignancies, both accredited as centre of excellence for ovarian cancer surgery by the European Society of Gynaecologic Oncology (ESGO).

Patients’ and tumour related characteristics including age, body-mass index (BMI), FIGO stage at diagnosis^7^, interval time to first recurrence and between two relapses, sites of recurrence, surgical procedures and adjuvant treatments were assessed and recorded.

The procedures were explained to the patients together with the therapeutic alternatives, and their written informed consent was obtained. At primary surgery all patients had undergone in the past a total hysterectomy, bilateral salpingo-oophorectomy with peritoneal and omental staging, with any additional debulking procedures, as required, to achieve complete tumour clearance and accurate staging (via laparotomy or laparoscopy), if fertility was not a concern. Unilateral salpingo-oophorectomy with peritoneal staging and endometrial sampling/ curettage were performed in cases of a fertility-sparing approach.

All cytoreductive surgeries in the recurrent setting were carried out via laparotomy. All patients who were deemed operable at relapse were considered for and offered surgery. The cases who had at least six months of disease‐free interval from initial diagnosis were considered as recurrent. Oncologic follow up consisted of regular clinical and radiological assessment as per local practice.

Adjuvant systemic treatment was decided based on local practice and protocols as per the local tumour board. Paclitaxel 175 mg/m^2^ IV followed by carboplatin AUC 5–6 IV Day 1, every 21 days for 3–6 cycles or BEP (Bleomycin 30 units IV per week plus etoposide 100 mg/m^2^ IV daily on days 1–5 plus cisplatin 20 mg/m^2^ IV daily on days 1–5) every 21 days for 3–4 cycles were administered as adjuvant systemic chemotherapy. As adjuvant antihormonal treatment, letrozole 2.5 mg/day, anastrazole 1 mg/day, gosereline asetat 3.6 mg every 28 days, leuprolide asetat 3.75 mg every 28 days or tamoxifen 20 mg/day were given until recurrence of disease or unacceptable toxicity.

To minimise interaction effects and simplify interpretation of outcomes, patients with incomplete tumour resection, those with combination systemic treatments i.e. chemotherapy combined with antihormonal therapy and those with other concurrent pathology or malignancies such as endometrial (pre)-cancer conditions, were excluded from the present analysis.

Our primary objective was to compare DFS between the different adjuvant treatment modalities. Secondary outcomes were to define risk factors of recurrence among patients and tumour related characteristics such as age, BMI, FIGO stage at diagnosis, interval time to recurrences, number of the recurrence, previous fertility sparing surgery (FSS), lymphadenectomy, residual tumour at primary surgery, adjuvant therapy at primary surgery, lymph node involvement, sites and number of recurrent tumour lesions, peritoneal involvement and presence of ascites.

### Statistical analysis

The Statistical Package for the Social Sciences (SPSS Inc., Chicago, IL) 29.0 version was used for all statistical analyses. The time from the date of the recurrence surgery to the final follow-up visit was calculated as the follow-up period. Survival was calculated based on the day of recurrence surgery. The Kaplan–Meier method was used to estimate survival estimates, and survival was compared with the log-rank test. Cox regression models were used for multivariate analysis. The Kruskal Wallis test for categorical variables and the ANOVA for continuous variables were used to determine statistical significance in order to compare the characteristics of the patients in each of the three groups. A p-value less than 0.05 was accepted as significant. Due to the fact that there were no deaths during the follow-up period, we were unable to analyse the patients’ overall survival.

### Ethics approval

The present study was approved by the institutional review board. (Istanbul University Faculty of Medicine-Number E-29624016-050.99-518857).

## Results

### Patient and disease characteristics

**Figure 1:**
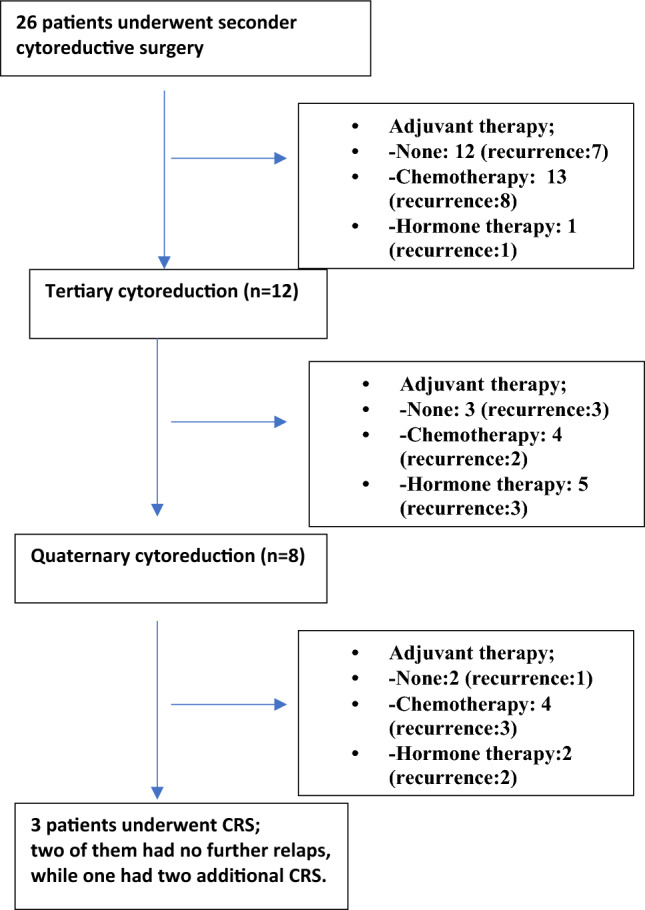
Study population.

We included 26 patients with relapsed GCT who underwent 51 cytoreductive surgeries resulting in total macroscopic tumour clearance over a 20-years period (2000–2020). The median age at initial diagnosis was 43 years (range 26–71). Twenty-six surgeries were for the 1st recurrence; 12 for the 2nd recurrence, 8 for the 3rd, 3 for the 4th recurrence and 2 for the 5th and 6th recurrence. Twelve (46%) of the twenty-six patients underwent multiple cytoreductive attempts for multiple recurrences. A flow chart is given in Fig. 1.

All patients had undergone primary surgery at initial diagnosis; 16 patients (61.5%) had a FIGO stage 1a. Detailed patients’ and initial treatment related characteristics are presented in Table 1. Only 2 patients had gross residual disease after primary cytoreduction. The majority of the patients were entered into only a watch and wait protocol at initial follow up without any 1st line adjuvant treatment.

**Table 1:** Patients’ and treatment related characteristics (n = 26).

Age at recurrence, years, median (range)	54 (33–75)
BMI in kg/m^2^, median (range)	33.1 (20.2–46.5)
Menopausal status at diagnosis	
Premenopause, n (%)	17 (65.4)
Postmenopause, n (%)	9 (34.6)
Initial stage (FIGO), n (%)	
1a	16 (61.5)
1c1	3 (11.5)
1c3	1 (3.9)
2b	1 (3.9)
3a	1 (3.9)
3c	4 (15.4)
Residual disease after primary surgery, n (%)	
Tumor free/optimal	24 (92.3)
Suboptimal	2 (7.7)
LND at initial surgery, n (%)	2 (7.7)
Initial fertility sparing surgery (FSS), n (%)	7 (26.9)
Adjuvant therapy after primary surgery, n (%)	
Cytotoxic chemotherapy (Carboplatin and taxol)	4 (15.4)
Antihormonal therapy	1 (3.9)
Chemotherapy + antihormonal therapy	1 (3.9)
Time from diagnosis to first relapse, in months, median (range)	83 (6–336)
Follow up period (from first relapse), months, median (range)	80 (12–193)

BMI: body-mass index LND: lymph node dissection FSS: fertility sparing surgery.

Median interval time from diagnosis to 1st -relapse was 83 months (range 6–336). After cytoreductive surgeries for the first, second and further recurrences, the recurrence rate was 61% (16/26), 66% (8/16) and 61% (8/13),respectively (Fig. 1). Sixteen patients experienced a 2nd recurrence after a median DFS of 36 months (range, 10–96) from the 1st recurrence. The median interval time between consecutive recurrences in all cases was 36 (range 6–76) months. The median disease-free interval from the treatment of the 2nd to the 3rd recurrence and 3rd to 4th recurrence were 30 and 36 months, respectively.

### Management and outcome following first recurrence

Surgical details of surgery at relapse are presented in Table 2. No adjuvant treatment was given in 20 (39.2%) recurrent cases. Cytotoxic chemotherapy was administered in 21 recurrences: 19 of them received carboplatin and paclitaxel and 2 were given a combination of bleomycin, etoposide, and cisplatin (BEP-regimen). Antihormonal treatment was applied in the other 10 recurrences: 5 cases received aromatase inhibitors (2 combined with GnRH agonists), 5 received tamoxifen (2 combined with GnRH agonists). Features of relapsed cases receiving antihormonal treatment or chemotherapy at relapse did not significantly differ from those receiving neither. After surgery for 1st-recurrence, median follow‐up of 26 patients was 80 months (range 12–193). At the time of last follow‐up, none of the patients had died from the disease. Twelve out of the 20 cases (60%) who underwent surgery alone experienced a further relapse during the follow up period of 80 months, versus 13/21(61.9%) of those cases who had surgery plus chemotherapy versus 7/10 (70%) of the cases who underwent surgery plus hormonal treatment (*p* = 0.616). There were no statistically significant differences in DFS between cases that received adjuvant chemotherapy, antihormonal therapy and those who did not receive any additional treatment.

**Table 2:** Operative and postoperative details of recurrence surgery (n = 51).

	n (%)
Location of tumor	
Pelvic	28 (54.9)
Upper abdomen	5 (9.8)
Both lower and upper abdomen	18 (35.3)
Number of tumor lesions	
One site	29 (56.9)
Oligo-metastatic (2–3)	12 (23.5)
Multi-focal	10 (19.6)
Lymph node involvement	
Pelvic	7 (13.7)
Paraaortic	3 (5.9)
Inguinal	2 (3.9)
Peritoneal spread (peritonectomy performed)	6 (11.8)
Ascites	4 (7.8)
Anterior wall of abdomen/rectus sheeth metastasis	7 (13.7)
Surgical procedures performed	
Ureteroneocystostomy	1 (1.9)
Large bowel resection	4 (7.8)
Small bowel resection	4 (7.8)
Ileostomy formation	0
Colostomy formation	1 (1.9)
Partial liver resection	1 (1.9)
Liver capsule resection	4 (7.8)
Adjuvant therapy at relapse	
None	20 (39.2)
Chemotherapy	21 (41.2)
Hormone therapy	10 (19.6)

**Figure 2:**
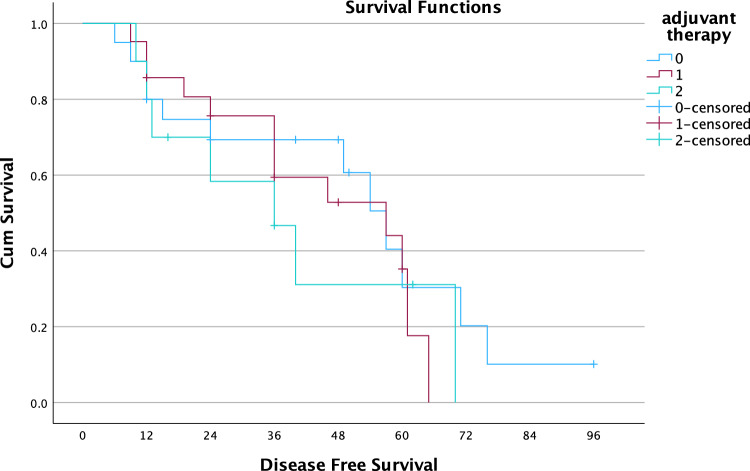
Kaplan–Meier survival curve for adjuvant therapy. 0: no adjuvant ,1: chemotherapy, 2:hormone therapy. Median DFS: 57 months (95% CI 45.9–68.0), 57 months (95% CI 23.2–90.8), 36 months (95% CI 16.9–55.1),respectively, log-rank p:0.616. Chemotherapy HR:1.296 (95% CI 0.559–3.002). Hormone therapy HR: 1.592 (95% CI 0.603–4.198).

Median DFS for patients who received adjuvant chemotherapy (n = 21) was 57 months (95% CI 23.2–90.8) versus 36 months (95% CI 16.9–55.1) for the patients who received adjuvant antihormonal therapy (n = 10) versus 57 months (95% CI 45.9–68.0) for the patients who did not receive any adjuvant treatment (n = 20) (*p* = 0.616). Kaplan–Meier curves are shown in Fig. 2.

In a subset analysis of patients with 1st-relapse (n = 26), excluding the one patient who received only antihormonal therapy, there was again no difference in the number of further recurrences between those who did (8/13 of whom 61.5% relapsed) and did not receive cytotoxic chemotherapy (7/12 of whom 58.3% relapsed). Moreover, adjuvant chemotherapy (n = 13) did not significantly prolong time to 2nd-recurrence (median DFS: 46 months (95% CI 24.6–67.4) versus those who did not receive any chemotherapy (n = 12) (57 months; 95% CI 49.7–64.4), *p* = 0.258.

### Predictors of disease free survival

**Figure 3:**
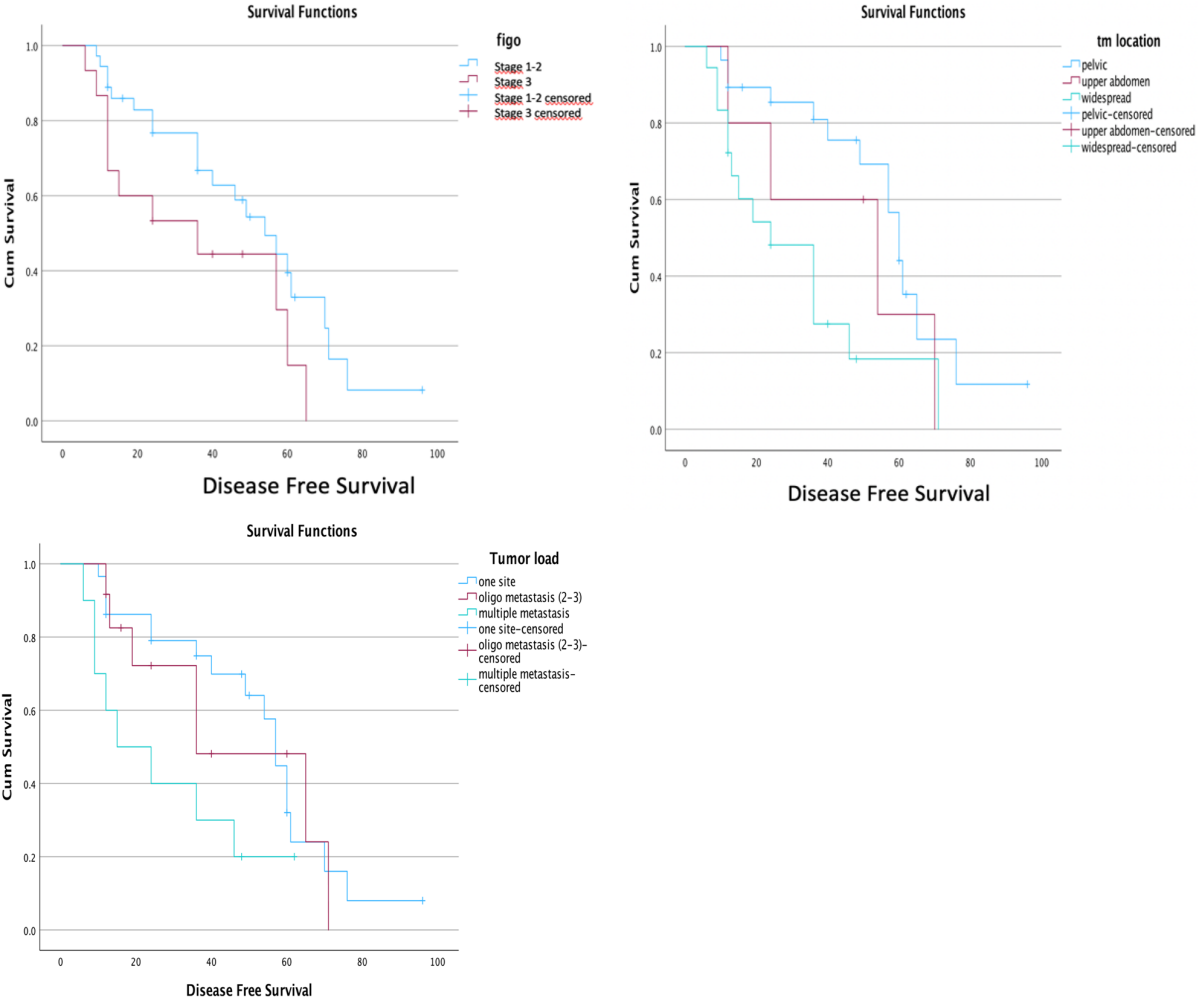
Kaplan–Meier survival curves.

When prognostic factors for DFS following complete cytoreductive surgery of 1st recurrence were examined by univariate and multivariate analysis, the site of the relapsed tumour (*p* = 0.007 and *p* = 0.009, respectively), the number of lesions at relapse (*p* = 0.049 and *p* = 0.058, respectively) and the initial FIGO stage (1–2 versus 3) (*p* = 0.06 and *p* = 0.036, respectively) were found to have predictive value for subsequent recurrences (Detailed data is given in Table 3 and Kaplan–Meier survival curves are shown in Fig. 3).

**Table 3:** Univariate and Multivariate analysis.

Characteristics	n	Median DFS (95%CI) months	*p* (log-rank)	Multivariate cox regression		
				HR	95% CI	*p*
FIGO stage						
1–2	36	54 (39.4–68.6)	0.06			0.036
3	15	36 (0.4–71.6)		3.07	1.34–7.04	
Tumor location						
Pelvic	28	60 (54.4–65.6)				
Upper abdomen	5	54 (7.2–100.8)	0.007	2.25	0.70–7.20	0.009
Both lower and upper abdomen	18	24 (8.8–39.2)		3.51	1.57–7.82	
Number of tumor lesions						
Single site	27	57 (51.7–62.3)				
Oligo-metastasis (2–3)	13	36 (4.8–67.2)	0.049	1.18	0.48–2.88	0.058
Multi-metastasis	11	15 (0–33.6)		2.74	1.15–6.54	

*Advanced age > 60 years, high BMI > 30 kg/m^2^, repeated recurrences, fertility preservation, residual tumour at initial surgery, lymphadenectomy at primary staging, adjuvant therapy after primary surgery, interval time to recurrence, ascites, lymph node involvement and peritoneal involvement at recurrence were not shown to significantly affect risk of relapse.

In univariate analysis, the following factors were not found to significantly affect DFS: advanced age > 60 years (n = 14) (*p* = 0.57), high BMI > 30 kg/m^2^ (n = 24) (*p* = 0.17), repeated recurrence (n = 25) (*p* = 0.67), fertility preservation (n = 7) (*p* = 0.32), residual tumour at initial surgery (n = 7) (*p* = 0.57), lymphadenectomy at primary staging (n = 2) (*p* = 0.31), adjuvant therapy after primary surgery (n = 14) (*p* = 0.79), interval time to recurrence (*p* = 0.64), ascites (n = 4) (*p* = 0.71), lymph node involvement (n = 12) (*p* = 0.65) and peritoneal involvement at recurrence (n = 6) (*p* = 0.27).

The most frequent location of recurrence was the pelvis (28/51, 54.9%). Recurrence was widespread in the abdomen in 35.3% (18/51) of cases. The twelve cases (23.5%) with lymph node relapse were all found to have metastases also at other sites: widespread + nodal (n = 7); upper abdominal organs and peritoneum + nodal (n = 2); and pelvic peritoneum and organs + nodal (n = 3). Even though site of recurrent disease appeared to significantly affect DFS, well established risk factors such as ascites (n = 4) and nodal involvement (n = 12) at relapse were not identified as predictors of reduced DFS (HR = 1.19, 95% CI 0.78–2.83, *p* = 0.71 and HR = 0.82, 95% CI 0.35–1.93, *p* = 0.65, respectively). None of the patients with lymph node involvement (n = 12) at recurrence had undergone lymph node dissection (LND) at primary surgery. In the 2 of the 26 patients who underwent LND at initial staging had negative LN status and did not relapse with nodal disease.

### Survival analyses for adjuvant therapy in advanced disease of GCT-relapse

The 18 cases who had tumor involvement in both the lower and upper abdomen (8 were oligometastatic, 4 were multi-focal without peritoneal carcinomatosis and 6 were multi-focal with diffuse peritoneal carcinomatosis) were further analyzed, and we found that 9 patients had received adjuvant chemotherapy after complete cytoreduction and 7 of them (77.8%) had experienced relapse. All patients who received antihormonal therapy relapsed (n = 2, 100%) and 5/7 (71.4%) of those who received no adjuvant treatment relapsed. Median DFS of the patients who had adjuvant chemotherapy (n = 9), antihormonal therapy (n = 2) and no adjuvant treatment (n = 7) were 36, 13 and 15 months respectively (*p* = 0.9). In the cases with only pelvic relapse who received adjuvant chemotherapy, 6/12 (50%) relapsed, while 2/5 (40%) of those with antihormonal therapy relapsed and 6/11 (54.5%) of those without any adjuvant treatment relapsed. Median DFS were 61, 40 and 57 months, respectively (*p* = 0.93).

For those with upper abdominal dissemination, all of those who received adjuvant antihormonal therapy relapsed (3/3) while 1/2 (50%) of those without any adjuvant treatment relapsed. Median DFS were 24 and 54 months, respectively. P value was not applicable because there was no case chemotherapy administered.

## Discussion

There is sufficient evidence indicating that maximal effort cytoreductive surgery is the cornerstone of treatment for primary and relapsed GCT with patients showing excellent outcomes despite repeated surgical procedures. Although postoperative adjuvant therapy is often administered to patients with relapsed disease, it is challenging to conduct well-designed randomized studies due to the rarity of the condition and significant differences in local practice and protocols^2,6^. Small retrospective series and case reports have demonstrated that platinum-based chemotherapy may induce tumour responses. Moreover, as a result of their high estrogenic activity, ovarian GCT are ideal candidates for antihormonal therapies such as tamoxifen, aromatase inhibitors and GnRH agonists. The use of chemotherapy or antihormonal therapies in patients with unresectable metastatic disease or those with suboptimal surgical outcomes is common practice, however limited data exist on the value of adjuvant systemic approaches following complete cytoreductive surgery in the relapsed setting^1,8, 9^. The 6-month clinical benefit rate of leuprolide acetate treatment of macroscopic disease was 66% in a cohort of 62 patients with recurrent granulosa cell tumors, and progression-free survival was similar to individuals receiving chemotherapy^10^. 13% of these were adjuvant to cytoreductive surgery.

According to ESGO guidelines, surgery is the preferred treatment option for patients with GCT-relapse. If complete debulking is achieved and the patient has not previously received chemotherapy, surveillance or adjuvant chemotherapy are offered as possible subsequent management options. If the patient has previously received chemotherapy, then the first option of choice following relapsed surgery is surveillance, and the second is adjuvant chemotherapy or antihormonal therapy^5^. The NCCN guidelines also include secondary cytoreductive surgery as preferable for relapse, but do not formulate concrete treatment recommendations regarding optimal systemic adjuvant options^3^.

We showed in this bicentric analysis over a long period of two decades, that no benefit was derived from adjuvant systemic treatment, of any type, following complete cytoreductive surgery in patients with GCT-relapse. Interestingly, we noted a trend of more favorable outcome in those patients who received cytotoxic chemotherapy when tumour dissemination patterns were multifocal, even if tumor-free following surgery. No significant value could be reached in this small series. Nevertheless, our findings may set the basis of future larger scale retrospective or even prospective randomised studies to evaluate the value of cytotoxic chemotherapy in the specific cohort of patients with high-risk relapse features.

Moreover, we identified extra-pelvic and/or multifocal tumor dissemination patterns at relapse, as well as advanced FIGO stage at initial diagnosis as independent risk factors for reduced remission after relapse surgery. Interestingly, we failed to attribute any significant prognostic value in well-established clinic-pathologic factors such as advanced age, high BMI, repeated recurrences, procedures and outcome of initial surgery, 1st line treatment modalities, interval time to recurrence, and unfavorable factors such as ascites, nodal and peritoneal involvement at recurrence.

Our findings correspond with the experience of other authors, even though in most studies, relapse GCT-patients are clustered together regardless of their surgical outcome. A retrospective study of 118 patients by Memorial Sloan Kettering showed that chemotherapy did not improve the recurrence-free interval of patients with GCTs (HR 0.98; *p* = 0.965), even though also non tumor-free operated patients were included^11^.

The multicentre retrospective MITO-9 study evaluating data on 35 recurrent GCTs observed further relapses in 33% of the patients who underwent surgery alone versus in 37.5% of the patients who had a combination approach with secondary cytoreduction surgery followed by chemotherapy as a treatment package. They identified only postoperative residual tumour as the only risk factor for reduced survival and so concluded that patients with repeated cytoreductive surgeries should avoid the toxicity of unnecessary chemotherapy especially if operated tumor-free^12^.

A research that examined 21 relapsed GCT cases in 8 patients suggested that adjuvant therapy may be of benefit in special higher risk situations, and that prognostic algorithms need to be identified at relapse to individualise treatment approaches and help to decide which cases would benefit from combination regimens^13^. Similar to our experience, they found that the number of tumour lesions, was a predictor of subsequent recurrence even when a total resection had been performed, and encouraged consideration of adjuvant chemotherapy for the sub-cohort of patients with multifocal and diffusely disseminated relapse. In a different study, adjuvant chemotherapy and total resection were shown to be effective, with 23 out of 40 cases being able to undergo complete resection. There was no significant difference in the regimens that were given. Nevertheless, the statistical analysis was conducted on 31 patients who had surgery whether it was optimal or suboptimal and 3 patients who had surgery without adjuvant chemotherapy^14^. They stated, in contrast to us, that the patients' survival was unaffected by the location of recurrence or multifocality.

According to a recent meta-analysis, whether the patients had early or advanced/recurrent disease, an adjuvant chemotherapy administration did not improve the oncological and prognostic results of GCT. Six of the 33 studies that addressed advanced and recurrent patients underwent subgroup analysis (OR 0.78), however it was unclear if total resection was carried out in these studies^15^.

In a retrospective analysis of seventy GCT patients with recurrent disease, 47 (67.1%) had adjuvant chemotherapy, while 10 (14.3%) had hormone treatment. In 57 individuals (81.4%), complete cytoreduction was performed. Adjuvant chemotherapy was found to be ineffective compared to those who did not receive it, however hormone therapy was not analyzed^16^.

Recurrent GCTs seem to tend to infiltrate the upper abdomen more often than in the primary setting^2^. In our analysis, advanced stage at initial presentation and widespread disease at relapse were independently associated with a less favorable DFS after surgery for relapse and it seemed that this was the cohort that may derive a clinical benefit from additional adjuvant cytotoxic chemotherapy after complete cytoreduction, but more conclusive evidence is of course needed.

Patients with single site or oligometastatic relapse and those with relapse limited to the pelvis seem to derive no benefit at all from any additional treatment approach and have an excellent survival after complete cytoreduction alone.

None of our patients died during our median follow-up period of 80 months, despite the chronic situation of their disease. Shim SH et al.^17^ also reported a 5-year OS of 100% after total debulking at a median follow-up of 50 (4–185) months following the initial recurrence, demonstrating once more that GCT patients have a different treatment profile than epithelial OC patients and do very well over decades with repeated cytoreductive attempts^2^.

Our study has the well-known disadvantages of any retrospective design. Moreover, adjuvant treatment decisions were based on the discretion of the treating physicians and on local protocols without any defined algorithms. Despite, hormone receptor status does not really predict response to hormone-based therapies^9,18, 19^, the lack of information about the hormonal receptor status of the tumour was another limitation. Due to the small sample size, it was difficult to achieve statistical significant differences and correlations.

A further significant gap is that we have missed reporting on the iatrogenic toxicity profiles of the different treatment strategies and we also did not present any quality of life data.

Despite the limitations of our research, our findings clearly support the current evidence that adjuvant therapy should not be routinely given in a recurrent CGT setting after complete cytoreduction. However, it seems that there is probably a sub-cohort of high-risk patients that will benefit from the combination of a surgical and systemic maximal effort approach, similar to the epithelial ovarian cancer population, and this deserves more research and consideration. Robust prognostic algorithms are warranted to maximise survival benefit after complete cytoreductive surgery and define best care.

Emerging promising targeted treatments for GCT patients will also be part of any future efforts. In 2014, a small phase 2 study revealed that single-agent bevacizumab, which targets vascular endothelial growth factor (VEGF), produced results in recurrent sex-cord stromal tumours (SCST) that showed a 17% response rate, 94% clinical benefit rate, and a median PFS of 9.3 months^20^. But, in an international randomized clinical trial of patients with relapsed SCSTs who were not candidates for surgery, adding bevacizumab to paclitaxel did not enhance therapeutic benefit^21^. There are signs that FOXL2 is a key player in the pathogenesis of adult GCT^22^ and may one day serve as a target for treatment, perhaps as an adjuvant.

## Conclusion

Our findings do not encourage the routine administration of adjuvant systemic therapy to patients who have undergone complete cytoreductive surgery for recurrent GCT. Maximal effort cytoreductive surgery remains the cornerstone of treatment also for relapsed disease. Selected high-risk patients with diffuse tumor dissemination at relapse may derive clinical benefit from additional cytotoxic chemotherapy following complete tumour clearance. We need as gynaecological oncology community to conduct larger scale multicentre studies to define robust prognostic and treatment algorithms for this rare disease.

## Data Availability

The datasets used and/or analysed during the current study available from the corresponding author on reasonable request.
